# A fractional-order approach to predator-prey interactions: Modeling fear and disease dynamics with memory effects

**DOI:** 10.1371/journal.pone.0339351

**Published:** 2026-02-02

**Authors:** Emli Rahmi, Nursanti Anggriani, Hasan S. Panigoro, Olumuyiwa James Peter

**Affiliations:** 1 Post Doctoral Program, Department of Mathematics, Universitas Padjadjaran, Kabupaten Sumedang, Jawa Barat, Indonesia; 2 Biomathematics Research Group, Department of Mathematics, Universitas Negeri Gorontalo, Bone Bolango, Indonesia; 3 Department of Mathematics, Universitas Padjadjaran, Kabupaten Sumedang, Jawa Barat, Indonesia; 4 Department of Mathematical and Computer Sciences, University of Medical Sciences, Ondo City, Ondo State, Nigeria; 5 Department of Epidemiology and Biostatistics, School of Public Health, University of Medical Sciences, Ondo City, Ondo State, Nigeria; Federal University of Technology - Parana, BRAZIL

## Abstract

The dynamical behaviors of a predator-prey model with fear effect and disease on prey are studied by employing the fractional-order derivative with a power-law kernel as the operator. The proposed model introduces four novel aspects: the impact of fear on a constant recruitment rate, previously unexplored in the literature; reinfection dynamics where infected prey can re-acquire the disease; selective predation by predators on infected prey due to pathogen-induced vulnerability; and the used of Caputo fractional derivative to include the memory effect. A deterministic approach is provided to establish the mathematical model including its validity by showing the existence, uniqueness, non-negativity, and boundedness. Three types of equilibrium points are obtained which represent the extinction of disease and predator, the predator-free, and the co-existence points. The local dynamics are investigated using Matignon’s condition and generalized Routh-Hurwitz criterion for the Caputo fractional-order model. The Volterra, quadratic, and linear functions are utilized to construct the Lyapunov function, as well as the LaSalle invariance principle, to show global dynamics. Some phenomena are provided namely forward and Hopf bifurcations to show the change of the dynamics when a parameter is varied. These results are supported by numerical ways using the predictor-corrector scheme.

## 1 Introduction

In the prey-predator interaction, predation could be seen as direct killing (lethal effects) or indirect killing (nonlethal effects). The study on predation as direct killing in prey-predator systems has been widely published since Lotka and Volterra proposed the first prey-predator model considering various functional responses; see, for example, [1–4]. However, some experimental investigations show that the indirect killing risk, also known as the fear effect, is sometimes more crucial than direct killing [5–7]. Zanette et al. [8] have manipulated song sparrows during an entire breeding season and found that the song sparrows’ reproductive success was reduced to 40% only by broadcasting predator sounds around all protected nests. Fear of predators caused the female song sparrows to lay fewer eggs affecting the birth rate, hatch fewer of those eggs (related to survival rate), and lose more nestlings to death in the nest (related to survival rate). Other evidence given by Elliott, et al. [9] which conducted an observation on the common fruit fly (*Drasophila melanogaster*) with the scent of its predator, mantid (*Tenodera sinensis*). The results show that by the end of the non-breeding season, the flies lost 60 % more mass and had a 64 % higher mortality rate while in the breeding season, they produced 20 % fewer offspring with a 9 % lower maturity weight when compared to the control. Moreover, how fear can suppress the reproductive output and offspring quality of species can also be found in wild rodents [10], female mice [11], Brandt’s voles [12], mountain goat [13], and bugs [14]. Based on all of this evidence, it is necessary to include both the direct and indirect killing effects of predators to characterize the dynamics of prey-predator interaction.

In 2016, Wang et al. [15] formulated the first predator-prey model that investigated the cost of fear in the prey population. They showed that high levels of fear could stabilize the predator-prey system by ruling out the existence of periodic solutions, whereas low levels of fear could create multiple limit cycles. The same result was also performed by Sarkar and Khajanchi [1]. Pal et al. [16] developed and analyzed a predator-prey model with hunting cooperation among predators that induces fear in the prey population. They showed how an increase in hunting cooperation might cause the system to destabilize and lead to periodic solutions through a Hopf-bifurcation. Moreover, Das and Samanta [17] work on the dynamical behavior of the prey-predator model with fear effect and Holling type IV functional response incorporating the memory effect. Analytical results show the local stability with the occurrence of saddle-node and Hopf bifurcation. Wu et al. [18] investigated the Leslie-Gower model incorporating the fear effect and nonlinear harvesting on prey species, and dynamical analysis shows the complex results, such as the existence of saddle-node, Hopf and Bogdanov-Taken bifurcation. Generally, the prey-predator models under consideration can be stabilized or destabilized by the fear effect.

Recently, the evolution of mathematical modeling in the context of prey-predator interactions and how pathogen transmission affects one [19,20] or both species [21,22] has become the interesting topic in mathematical biology. In ecological communities, the predators can either positively or negatively influence disease dynamics through their effects on prey density and traits [23]. The positive impact occurs when the pathogen in infected prey could infect the predator as well. Conversely, if predation selectively removes the infected prey without affecting the predator then there is the possibility to reduce the pathogen in the prey population [24,25]. In this case, disease has a negative effect on the dynamics of the predator-prey model. From the view of dynamical behavior, the spread of pathogens in a Lotka-Volterra predator-prey model causes the existing prey-predator cycle to be disrupted, and the interactions will become more complex [26].

The further exploration study of the intermingling between a prey-predator model incorporating fear effect and the spread of disease either in prey or predator population has gained widespread concern in order to protect endangered species. Sarkar and Khajanchi [27] construct the model by assuming that the prey population grows logistically in the absence of predator species and that predators feed both susceptible and infected prey. They revealed that increasing levels of fear could stabilize the system toward the equilibrium point from a limit cycle oscillation condition. In this case, the disease still exists in the system but the amplitude of the infected prey decreases. Further, as the rate of infection in prey increases to a certain level the predator becomes extinct though predators are not infected by the disease. Hossain, et al. [28] also investigated the impact of fear in an eco-epidemiological model which assumes the prey grows logistically. The transmission rate of disease in the prey population follows a general disease transmission term. Kashyap et al. [29] work on the scenario of how the fear response in prey with disease could affect the mass mortality of predators (case in the Salton sea between tilapia fish and pelican birds) using the Caputo fractional derivative. On the other hand, Barman et al. [30] presented the eco-epidemiological model with infected disease in predator populations whether the fear induced by susceptible and infected predators at different levels. Zhang [26] also established the prey-predator model with standard incidence term as the transmission mechanism of infectious disease in the predator population.

Motivated by the above literature, in this paper, we propose a model addressing these key contributions:

1. The impact of fear on the constant recruitment rate which never considered previous work given by Moustafa et al. [31].
2. Infected prey can be able to reinfection the disease.
3. Since infected prey typically have pathogen-induced morbidity that makes them more vulnerable to predation, predators exclusively feed the infected ones [32–34].
4. The Caputo fractional differential equation is employed to involve memory and hereditary properties which are nonlocal [35–40].

The following is the structure for the following content in this paper: Some preliminaries about the fractional calculus are recalled in Sect 2. In Sect 3, we formulate the eco-epidemiological model under the influence of an SIS infectious disease in the prey population by using the Caputo FDE. In Sect 4, we explore some qualitative analysis of the model by showing their biological validity, the existence of equilibrium points, local and global dynamics, and the occurrence of bifurcation phenomena. The numerical simulation of the model is given in Sect 5. We end the paper by summarizing the results in Sect 6.

## 2 Preliminaries

Some advantageous tools are provided below which consist of definitions, lemmas, and theorems related to fractional calculus and their application to mathematical modeling. We also propose some references such as [41–44] to obtain more in-depth information about the Caputo FDE.

**Definition 1.** [43] The Caputo FDE of order α∈(0,1] is defined by


$${\;}^{C}{𝒟}_{t}^{\alpha }z(t):=\frac{1}{\Gamma \left(1-\alpha \right)}\int_{0}^{t}\frac{z^{\prime }(\tau )}{{\left(t-\tau \right)}^{\alpha }}\;d\tau ,$$

where the function $z:C^{n}[0,\infty )\to \mathbb{R}$ with $C^{n}[0,\infty )$ is a space of *n* times continuously differentiable function and Γ(·) is the Gamma function.

**Lemma 1.** [45] Consider the following Caputo FDE.

$${\;}^{C}{𝒟}_{t}^{\alpha }\vec{z}(t)=\vec{w}(\vec{z}(t)),\;\vec{z}\in {\mathbb{R}}^{n},\;\alpha \in (0,1],$$ (1)

with initial condition $z_{t_{0}}$ and $w:\Phi \to {\mathbb{R}}^{n},\;\Phi \subset {\mathbb{R}}^{n}$. The Caputo FDE (1) has a unique solution in *Φ* when *w*(*z*,*t*) satisfies the local Lipschitz condition i.e


$$\left\|w(z)-w(\bar{z})\right\|\leq 𝒦\left\|z-\bar{z}\right\|,$$

for any $z,\bar{z}\in \Phi$ where 𝒦 is a Lipschitz constant.

**Theorem 1.** [46] *The Caputo FDE* (1) *has non-negative solution for any non-negative initial condition as* t→∞ *if (i) the function* $\vec{w}(\vec{z}(t))$ *satisfies locally Lipschitz condition; and (ii) for* α=1*, the Caputo FDE* (1) *has the non-negative solution.*

**Lemma 2.** [47] Let $z(t)\in C([t_{0},\infty ))$ satisfies


$${\;}^{C}{𝒟}_{t}^{\alpha }z(t)+\varphi z(t)\leq \bar{\varphi },\;t_{0}\geq 0,\;z(t_{0})=z_{t_{0}},\;\alpha \in (0,1],\;\left\{\varphi ,\bar{\varphi }\right\}\in \mathbb{R},\;\varphi \neq 0.$$

Then, the following inequality holds.


$$z(t)\leq \left(z_{t_{0}}-\frac{\bar{\varphi }}{\varphi }\right)E_{\alpha }[-\varphi (t-t_{0}{)}^{\alpha }]+\frac{\bar{\varphi }}{\varphi }.$$

**Theorem 2** (Matignon Condition [43,48]). *A point* ${\vec{z}}^{*}$ *is called the equilibrium point of the Caputo FDE* (1) *if* $\vec{w}({\vec{z}}^{*}(t))=0$*. Moreover, the equilibrium point* ${\vec{z}}^{*}$ *is said locally asymptotically stable (LAS) if all eigenvalues* $\lambda_{j}$ *of the Jacobian matrix* $𝒥={\frac{\partial \vec{w}}{\partial z}|}_{{\vec{z}}^{*}}$ *satisfy* $\left|\arg(\lambda_{j})\right|>\frac{\alpha \pi }{2}$*. It is said a saddle-point if there exist* {l,k}∈ℕ, l≠k *such that* $\left|\arg(\lambda_{k})\right|>\frac{\alpha \pi }{2}$ *and* $\left|\arg(\lambda_{l})\right|<\frac{\alpha \pi }{2}$.

**Theorem 3.** [49] *Consider the Caputo FDE* (1) *with n* = 3 *and the Jacobian matrix evaluated at the equilibrium point* ${\vec{z}}^{*}\in {\mathbb{R}}^{3}$ *has polynomial characteristic*

$$𝒫(\lambda )=\lambda^{3}+c_{1}\lambda^{2}+c_{2}\lambda +c_{3},\;a_{i}\in \mathbb{R},\;i=1,2,3,$$ (2)

*where has the discriminant* $\Delta (𝒫)=18c_{1}c_{2}c_{3}+(c_{1}c_{2}{)}^{2}-4c_{3}(c_{1}{)}^{3}-4(c_{2}{)}^{3}-27(c_{3}{)}^{2}$*. All eigenvalues of the Jacobian matrix given by the polynomial characteristic* (2) *satisfies* $\left|\arg(\lambda_{i})\right|>\frac{\alpha \pi }{2},\;i=1,2,3$ *if*

(i) Δ>0*, c*_1_>0*, c*_3_>0*, and* $c_{1}c_{2}>c_{3}$*, or*
(ii) Δ<0, $c_{1}\geq 0$, $c_{2}\geq 0$*, c*_3_>0 *and* α<2/3*, or*
(iii) Δ<0*, c*_1_>0*, c*_2_>0*, and* $c_{1}c_{2}=c_{3}$.

*If* Δ<0*, c*_1_<0*, c*_2_<0*, and* α>2/3 *then all eigen values satisfies* $\left|\arg(\lambda_{i})\right|<\frac{\alpha \pi }{2},\;i=1,2,3$.

**Lemma 3** (Quadratic-type Lyapunov functions [50]). For $z(t)\in C([t_{0},\infty ))$, the following inequality holds.


$${\;}^{C}{𝒟}_{t}^{\alpha }z^{2}(t)\leq 2z(t{)}^{C}{𝒟}_{t}^{\alpha }z(t),$$

for all α∈(0,1] and $t\geq t_{0}$.

**Lemma 4** (Volterra-type Lyapunov functions [51]). Let $z(t)\in {\mathbb{R}}^{+}$ and $\bar{z}\in {\mathbb{R}}^{+}$. For $z(t)\in C([t_{0},\infty ))$, the following inequality holds.


$${\;}^{C}{𝒟}_{t}^{\alpha }\left[z(t)-\bar{z}-\bar{z}\ln\frac{z(t)}{\bar{z}}\right]\leq (1-\frac{\bar{z}}{z(t)}{)}^{C}{𝒟}_{t}^{\alpha }z(t),$$

for all α∈(0,1] and $t\geq t_{0}$.

**Lemma 5.** [52] Suppose that *Φ* is a bounded closed set and every solution of the Caputo FDE (1) which has the initial condition in *Φ* for t→∞. If there exists 𝒱(z):Φ→ℝ with continuous first order partial derivatives satisfies


$${{\;}^{C}{𝒟}_{t}^{\alpha }𝒱|}_{eq.(1)}\leq 0,$$

then every solution *z*(*t*) originating in Ψ tends to ℰ as t→∞, where ℰ is the largerst invariant set of $ℳ:=\left\{z:{{\;}^{C}{𝒟}_{t}^{\alpha }𝒱|}_{eq.(1)}=0\right\}$. If ℰ is the equilibrium point of eq. 1 which satisfies this condition, then ℰ is said globally asymptotically stable (GAS).

## 3 Model formulation

In this paper, we construct the prey-predator model where the prey population follows the SIS (Susceptible-Infected-Susceptible) type and the predator-induced fear effect on the prey population. We modify the eco-epidemic model proposed by Moustafa et al. [31]. The model assumes that there are two populations namely prey and predator where prey is divided into two compartments namely the susceptible and infected prey which respectively denoted by *S* and *I* and the predator denoted by *P* which only targeting the infected prey for food with Holling type-2 for the predatio functional response. As a result, we have the following model.

$$\frac{dS}{d\hat{t}}=\;A-d_{1}S-bSI+wI,\;\frac{dI}{d\hat{t}}=\;bSI-\left(d_{2}+w\right)I-\frac{cIP}{1+aI},\;\frac{dP}{d\hat{t}}=\;\frac{eIP}{1+aI}-d_{3}P,$$ (3)

where the biological interpretation of each variable and parameter is given in Table 1. To involve the fear effect, we multiply the recruitment rate into susceptible prey population by the fear factor term $f(k,P)=\frac{1}{1+kP}$ where the parameter *k* represents the level of fear that reduces the birth rate of prey [53,54]. As a result, we have

**Table 1:** Biological interpretation of variables and parameters of model (6).

Parameters	Biological interpretation
*A*	The constant recruitment rate of susceptible prey population corresponding to births and immigration
*b*	The infection rate of the disease in prey
*w*	The recovery rate of the disease in prey
*d*_1_	The mortality rate of susceptible prey
*d*_2_	The mortality rate of infected prey
*d*_3_	The mortality rate of predator
*c*	The predation rate on infected prey
*e*	The birth rate which converted from the predation process on infected prey
*a*	Half saturation constant
*k*	Level of fear that reduces the birth rate of prey

$$\frac{dS}{d\hat{t}}=\;\frac{A}{1+kP}-d_{1}S-bSI+wI,\;\frac{dI}{d\hat{t}}=\;bSI-\left(d_{2}+w\right)I-\frac{cIP}{1+aI},\;\frac{dP}{d\hat{t}}=\;\frac{eIP}{1+aI}-d_{3}P.$$ (4)

To include the memory effect to the model, we replace the first-order derivative at the left-hand side of model (4) with the fractional-order derivative $\frac{d^{\alpha }(\cdot )}{d{\hat{t}}^{\alpha }}$ along by rescale the dimension of time of the parameters to maintain their consistency. Therefore, we have

$$\frac{d^{\alpha }S}{d{\hat{t}}^{\alpha }}=\;\frac{A^{\alpha }}{1+kP}-d_{1}^{\alpha }S-b^{\alpha }SI+w^{\alpha }I,\;\frac{d^{\alpha }I}{d{\hat{t}}^{\alpha }}=\;b^{\alpha }SI-\left(d_{2}^{\alpha }+w^{\alpha }\right)I-\frac{c^{\alpha }IP}{1+aI},\;\frac{d^{\alpha }P}{d{\hat{t}}^{\alpha }}=\;\frac{e^{\alpha }IP}{1+aI}-d_{3}^{\alpha }P.$$ (5)

Using Caputo fractional derivative given by Definition 1 and rewriting the parameter values such that $\frac{d^{\alpha }S}{d{\hat{t}}^{\alpha }}={\;}^{C}{𝒟}_{t}^{\alpha }S$, $\frac{d^{\alpha }I}{d{\hat{t}}^{\alpha }}={\;}^{C}{𝒟}_{t}^{\alpha }I$, $\frac{d^{\alpha }P}{d{\hat{t}}^{\alpha }}={\;}^{C}{𝒟}_{t}^{\alpha }P$, $A^{\alpha }=\Lambda$, $d_{1}^{\alpha }=\mu$, $b^{\alpha }=\beta$, $w^{\alpha }=\omega$, $d_{2}^{\alpha }=\delta$, $c^{\alpha }=m$, $e^{\alpha }=n$, and $d_{3}^{\alpha }=d$, we have

$${\;}^{C}{𝒟}_{t}^{\alpha }S=\;\frac{\Lambda }{1+kP}-\mu S-\beta SI+\omega I=F_{1}(S,I,P),\;{\;}^{C}{𝒟}_{t}^{\alpha }I=\;\beta SI-\left(\delta +\omega \right)I-\frac{mIP}{1+aI}=F_{2}(S,I,P),\;{\;}^{C}{𝒟}_{t}^{\alpha }P=\;\frac{nIP}{1+aI}-dP=F_{3}(S,I,P).$$ (6)

In the next section, we will study the validity of the model, the dynamical behaviors, and some exploration in numerical simulations.

## 4 Qualitative analysis

### 4.1 Biological validity

We present this subsection to give the biological validity of the model by showing that each solution given by model (6) with non-negative initial condition will always exists, unique, non-negative, and bounded in the biological region


$${\mathbb{R}}_{+}^{3}:=\left\{(S,I,P)\;|\;S\geq 0,\;I\geq 0,\;\text{and}\;P\geq 0,\;\left\{S,I,P\right\}\in \mathbb{R}\right\}.$$

We provide the following theorems to show the existence, uniqueness, non-negativity, and boundedness of the solution of model (6).

**Theorem 4.** *Let*


$$\Phi :=\left\{(S,I,P)\in {\mathbb{R}}_{+}^{3}:\max\left\{\left|S\right|,\left|I\right|,\left|P\right|\right\}\leq ℳ\right\},\;ℳ>0$$

*Model* (6) *with initial condition in Φ has unique solution in* $[t_{0},\infty )\times \Phi$.

*Proof*: Let 𝒳=(S,I,P) and $\bar{𝒳}=\left(\bar{S},\bar{I},\bar{P}\right)$ are solutions of model (6). For $ℋ(𝒳)=\left(F_{1}(𝒳),F_{2}(𝒳),F_{3}(𝒳)\right)$, we have


$$\left\|ℋ(𝒳)-ℋ(\bar{𝒳})\right\|=\;\left|F_{1}(𝒳)-F_{1}(\bar{𝒳})\right|+\left|F_{2}(𝒳)-F_{2}(\bar{𝒳})\right|+\left|F_{3}(𝒳)-F_{3}(\bar{𝒳})\right|$$

$$=\;\left|\left(\frac{\Lambda }{1+kP}-\mu S-\beta SI+\omega I\right)-\left(\frac{\Lambda }{1+k\bar{P}}-\mu \bar{S}-\beta \bar{S}\bar{I}+\omega \bar{I}\right)\right|$$

$$\;+\left|\left(\beta SI-\left(\delta +\omega \right)I-\frac{mIP}{1+aI}\right)-\left(\beta \bar{S}\bar{I}-\left(\delta +\omega \right)\bar{I}-\frac{m\bar{I}\bar{P}}{1+a\bar{I}}\right)\right|$$

$$\;+\left|\left(\frac{nIP}{1+aI}-dP\right)-\left(\frac{n\bar{I}\bar{P}}{1+a\bar{I}}-d\bar{P}\right)\right|$$

$$=\;|-\frac{\Lambda k}{\left(1+kP)(1+k\bar{P}\right)}\left(P-\bar{P}\right)-\left(\mu +\beta I\right)\left(S-\bar{S}\right)$$

$$\;-\left(\beta \bar{S}-\omega \right)\left(I-\bar{I}\right)+|\beta I\left(S-\bar{S}\right)+\left(\beta \bar{S}+\delta +\omega \right)\left(I-\bar{I}\right)$$

$$\;-\frac{mP(I-\bar{I})}{\left(1+aI)(1+a\bar{I}\right)}-\frac{m\bar{I}\left(P-\bar{P}\right)}{1+a\bar{I}}|+|\frac{nP\left(I-\bar{I}\right)}{\left(1+aI)(1+a\bar{I}\right)}+\frac{n\bar{I}\left(P-\bar{P}\right)}{1+a\bar{I}}$$

$$\;-d\left(P-\bar{P}\right)|$$

$$\leq \;\Lambda k\left|P-\bar{P}\right|+\left(\mu +\beta ℳ\right)\left|S-\bar{S}\right|+\left(\beta ℳ+\omega \right)\left|I-\bar{I}\right|$$

$$\;+\beta ℳ\left|S-\bar{S}\right|+\left(\beta ℳ+\delta +\omega \right)\left|I-\bar{I}\right|+mℳ\left|I-\bar{I}\right|$$

$$\;+mℳ\left|P-\bar{P}\right|+nℳ\left|I-\bar{I}\right|+nℳ\left|P-\bar{P}\right|+d\left|P-\bar{P}\right|$$

$$=\;\left(\mu +2\beta ℳ\right)\left|S-\bar{S}\right|+\left(\left(2\beta +m+n\right)ℳ+2\omega +\delta \right)\left|I-\bar{I}\right|$$

$$\;+\left(\Lambda k+d+\left(m+n\right)ℳ\right)\left|P-\bar{P}\right|$$

$$\leq \;ℒ\left|𝒳-\bar{𝒳}\right|,$$

where ℒ:=max{(μ+2βℳ),((2β+m+n)ℳ+2ω+δ)+(Λk+d+(m+n)ℳ)}. According to Lemma 1, the function ℋ(𝒳) satisfies the locally Lipschitz condition with respect to the variables 𝒳=(S,I,P) with ℒ is the Lipschitz constant. Thus, the model (6) has the unique solution in $[t_{0},\infty )\times \Phi$ for each initial condition in *Φ*. □

**Theorem 5.** *The solution S(t), I(t), and P(t) of model* (6) *always stays on* ${\mathbb{R}}_{+}^{3}$ *for any* S(0)≥0, I(0)≥0, P(0)≥0*, and* t→∞.

*Proof*: For α=1, model (6) can be rewritten as follows.

$$\frac{dS}{dt}=\;\frac{\Lambda }{1+kP}+\omega I-G_{1}S,\;\frac{dI}{dt}=\;G_{2}I,\;\frac{dP}{dt}=\;G_{3}P,$$ (7)

where $G_{1}=\mu +\beta I$, $G_{2}=\beta S-\left(\delta +\omega \right)-\frac{mP}{1+aI}$, and $G_{3}=\frac{nI}{1+aI}-d$. From the second and the third equations on (7), we get solutions


$$I(t)=\;I(0)e^{\int_{0}^{t}G_{2}\;d\tau },$$

$$P(t)=\;P(0)e^{\int_{0}^{t}G_{3}\;d\tau }.$$

Since the initial conditions I(0)≥0 and P(0)≥0, we confirm that I(t)≥0 and P(t)≥0 as t→∞. From the non-negativity of *I*(*t*) and *P*(*t*), we derive the first equation on (7) as


$$\frac{dS}{dt}=\;\frac{\Lambda }{1+kP}+\omega I-G_{1}S,$$

$$\geq \;-G_{1}S.$$

Thus, we have $S(t)\geq S(0)e^{-\int_{0}^{t}G_{1}\;d\tau }$ which always non-negative since I(0)≥0. Furthermore, based on the proof of Theorem 4, we also have ℋ(𝒳) satisfies the locally Lipschitz condition with respect to the variables 𝒳=(S,I,P). We assure from Theorem 1 that the model (6) always stays on ${\mathbb{R}}_{+}^{3}$ for any non-negative initial condition. □

**Theorem 6.** *Let* $\left\{S(0),I(0),P(0)\right\}\in {\mathbb{R}}_{+}^{3}$*. The solution of model* (6) *will always bounded.*

*Proof*: We define the positive function as follows.

$$𝒜=S+I+\frac{m}{n}P.$$ (8)

by computing the Caputo fractional derivative of eq. 8, we obtain


$${\;}^{C}{𝒟}_{t}^{\alpha }N=\;{\;}^{C}{𝒟}_{t}^{\alpha }S+{\;}^{C}{𝒟}_{t}^{\alpha }I+\frac{m}{n}{\;}^{C}{𝒟}_{t}^{\alpha }P$$

$$=\;\left(\frac{\Lambda }{1+kP}-\mu S-\beta SI+\omega I\right)+\left(\beta SI-\left(\delta +\omega \right)I-\frac{mIP}{1+aI}\right)$$

$$\;+\frac{m}{n}\left(\frac{nIP}{1+aI}-dP\right)$$

$$=\;\frac{\Lambda }{1+kP}-\mu S-\delta I-\frac{dmP}{n}.$$

Let 0<θ≤min{μ,δ,d}. we achieve


$${\;}^{C}{𝒟}_{t}^{\alpha }𝒜+\theta 𝒜=\;\frac{\Lambda }{1+kP}-\mu S-\delta I-\frac{dmP}{n}+\theta S+\theta I+\frac{\theta m}{n}P$$

$$=\;\frac{\Lambda }{1+kP}-\left(\mu -\theta \right)S-\left(\delta -\theta \right)I-\frac{m}{n}\left(d-\theta \right)P$$

≤ Λ,

and hence we have ${\;}^{C}{𝒟}_{t}^{\alpha }𝒜+\theta 𝒜\leq \Lambda$. According to Lemma 2, the following inequality holds.


$$𝒜(t)\leq \left({𝒜}_{t_{0}}-\frac{\Lambda }{\theta }\right)E_{\alpha }[-\theta (t-t_{0}{)}^{\alpha }]+\frac{\Lambda }{\theta },$$

where *E* is the Mittag-Leffler function. According to the convergence of the Mittag-Leffler function [43], we have $𝒜(t)\to \frac{\Lambda }{\theta }$ as t→∞. Thus, for any non-negative initial condition, the solution is confined to 𝒰 where

$$𝒰:=\left\{(S,I,P)\in {\mathbb{R}}_{+}^{3}:𝒜(t)<\frac{\Lambda }{\theta }+\varepsilon =\gamma ,\;\varepsilon >0\right\}.$$ (9)

This completes the proof. □

### 4.2 Feasible equilibrium points

In this subsection, all feasible equilibrium points are investigated including their existence condition. Following Theorem 2, the equilibrium points are obtained by solving $F_{1}=F_{2}=F_{3}=0$ as well as finding the positive solutions of the following equations.

$$\frac{\Lambda }{1+kP}-\mu S-\beta SI+\omega I=\;0,\;\left[\beta S-\left(\delta +\omega \right)-\frac{mP}{1+aI}\right]I=\;0,\;\left[\frac{nI}{1+aI}-d\right]P=\;0.$$ (10)

As a result, we have three equilibrium points as follows.

(i) The prey-disease-predator-free point (PDPF) given by
  $${ℰ}_{1}=\left(\frac{\Lambda }{\mu },0,0\right),$$ (11)
  which describes the condition where only the susceptible prey exists in the ecosystem while the disease disappears and the predator is extinct. To include the impact of the spread of the disease on prey, we compute the basic reproduction number $\left({ℛ}_{0}\right)$ using the next generation matrix as in [55,56]. We first consider the new infection term $\left(\Delta_{F}\right)$ and the remaining transfer terms $\left(\Delta_{V}\right)$ given by the second equation on (6) as follows.
  $$\Delta_{F}=\beta SI,\;\text{and}\;\Delta_{V}=(\delta +\omega )I+\frac{mIP}{1+aI}.$$
  Therefore, we have the basic reproduction number by solving $\Delta_{F}\Delta_{V}^{-1}$ at ${ℰ}_{1}=\left(\frac{\Lambda }{\mu },0,0\right)$. The following term is acquired.
  $${ℛ}_{0}=\;\Delta_{F}\Delta_{V}^{-1}\;=\;{\frac{\beta S}{(\delta +\omega )+\frac{mP}{(1+aI{)}^{2}}}|}_{{ℰ}_{1}}\;=\;\frac{\beta \Lambda }{\left(\delta +\omega \right)\mu }.$$ (12)
(ii) The predator-free point (PFP) represents the condition when only susceptible and infected prey exists in the ecosystem while the predator becomes extinct. The PFP is defined by
  $${ℰ}_{2}=\left(\frac{\Lambda }{\mu {ℛ}_{0}},\frac{\left({ℛ}_{0}-1\right)\Lambda }{\delta {ℛ}_{0}},0\right).$$ (13)
(iii) The co-existence point (CEP) is given by ${ℰ}_{3}=\left(\eta ,\sigma ,\kappa \right)$ where $\sigma =\frac{d}{n-ad}$, $\eta =\frac{\Lambda }{\mu {ℛ}_{0}}+\frac{m\kappa }{(1+\sigma a)\beta }$, and κ is the positive root respect to *P* of the quadratic equation $\zeta_{1}P^{2}+\zeta_{2}P+\zeta_{3}=0$ with
  $$\zeta_{1}=\;\left(\beta \sigma +\mu \right)km,$$
$$\zeta_{2}=\;\left(\delta \sigma +\frac{\Lambda }{{ℛ}_{0}}\right)\left(1+\sigma a\right)\beta k+\left(\beta \sigma +\mu \right)m,$$
$$\zeta_{3}=\;\left({ℛ}_{0}-\frac{\Lambda }{\Lambda -\delta \sigma }\right)\frac{\left(\Lambda -\delta \sigma \right)\left(1+\sigma a\right)\beta \Lambda }{{ℛ}_{0}},$$
  which describes the condition when susceptible prey, infected prey, and predator exist in the ecosystem.

The existence conditions for each equilibrium point are given by the following theorem.

**Theorem 7.** *For each equilibrium point* ${ℰ}_{i},\;i=1,2,3$, *we have*

(i) *The PDPF* ${ℰ}_{1}$ *is always exists;*
(ii) *The PFP* ${ℰ}_{2}$ *exists only if* ${ℛ}_{0}>1$*; and*
(i) *The CEP* ${ℰ}_{3}=\left(\eta ,\sigma ,\kappa \right)$ *exists and unique if n*>*ad and: (i)* $\sigma >\frac{\Lambda }{\delta }$*, or (ii)* $\sigma <\frac{\Lambda }{\delta }$ *and* ${ℛ}_{0}<\frac{\Lambda }{\Lambda -\delta \sigma }$.

*Proof*: It is clear that the PDPF always exists since ${ℰ}_{1}\in {\mathbb{R}}_{+}^{3}$. For PFP, we emphasize that ${ℰ}_{2}\in {\mathbb{R}}_{+}^{3}$ only if ${ℛ}_{0}>1$. Next, the CEP ${ℰ}_{3}\in {\mathbb{R}}_{+}^{3}$ only when η>0, σ>0, and κ>0. We identify that $\zeta_{1}$ and $\zeta_{2}$ are always positive numbers. It is clear that σ>0 only when *n*>*ad*. Moreover, η>0 when κ>0. Thus, the existence of CEP depends on the value of κ. By identifying the positive root of *P*, we obtain


$$P_{1}=\;-\frac{1}{2}\left[\zeta_{2}+\sqrt{\zeta^{2}-4\zeta_{1}\zeta_{3}}\right],$$

$$P_{2}=\;-\frac{1}{2}\left[\zeta_{2}-\sqrt{\zeta^{2}-4\zeta_{1}\zeta_{3}}\right].$$

Therefore, three conditions occur: (i) when $\zeta_{2}^{2}=4\zeta_{1}\zeta_{3}$, $P_{1}=P_{2}=-\frac{\zeta_{2}}{2}<0$ and CEP does not exist, (ii) when $\zeta_{2}<4\zeta_{1}\zeta_{3}$, both *P*_1_ and *P*_2_ become complex numbers and CEP does not exist, (iii) when $\zeta_{2}^{2}>4\zeta_{1}\zeta_{3}$, *P*_1_ always negative and *P*_2_<0 only when $\zeta_{3}<0$. When $\sigma >\frac{\Lambda }{\delta }$, the value of $\zeta_{3}$ always negative. When $\sigma <\frac{\Lambda }{\delta }$, $\zeta_{3}<0$ only when ${ℛ}_{0}<\frac{\Lambda }{\Lambda -\delta \sigma }$. This shows that only one equilibrium point may exist in the interior of the model (6). □

### 4.3 Local stability analysis

We study the dynamical behavior around the equilibrium point of model (6). The local stability theorem given by Matignon’s (see Theorem 2) is employed. Thus the following theorems are successfully established.

**Theorem 8.** *The PDPF is LAS if* ${ℛ}_{0}<1$ *and a saddle point if* ${ℛ}_{0}>1$.

*Proof*: Applying linearization, we have the following Jacobian matrix for ${ℰ}_{1}=\left(\frac{\Lambda }{\mu },0,0\right)$.

$${𝒥(S,I,P)|}_{{ℰ}_{1}}=[\begin{array}{ccc} -\mu & -\frac{\beta \Lambda }{\mu }+\omega & -\Lambda k\\ 0 & \left(\delta +\omega \right)\left({ℛ}_{0}-1\right) & 0\\ 0 & 0 & -d \end{array}].$$ (14)

Therefore, the following eigenvalues hold: $\lambda_{1}=\mu$, $\lambda_{2}=\left(\delta +\omega \right)\left({ℛ}_{0}-1\right)$, and $\lambda_{3}=-d$. Since $\lambda_{1,3}<0$, we confirm $\left|\mathrm{\backslash arg}\left(\lambda_{1,3}\right)\right|=\pi >\frac{\alpha \pi }{2}$ and hence the stability of ${ℰ}_{1}$ depends on the sign of $\lambda_{2}$. It is verified that $\left|\mathrm{\backslash arg}\left(\lambda_{2}\right)\right|>\frac{\alpha \pi }{2}$ when ${ℛ}_{0}<1$ and $\left|\mathrm{\backslash arg}\left(\lambda_{2}\right)\right|<\frac{\alpha \pi }{2}$ when ${ℛ}_{0}>1$. Following Matignon’s condition (see Theorem 2), the validity of the theorem has been proven. □

**Theorem 9.** *Let* $\xi_{1}=\frac{\left({ℛ}_{0}-1\right)\beta \Lambda }{\delta {ℛ}_{0}}$, $\xi_{2}=\frac{\left({ℛ}_{0}-1\right)\Lambda }{\delta {ℛ}_{0}+\left({ℛ}_{0}-1\right)\Lambda a}$*, and* $\xi_{3}=\sqrt{\mu^{2}+2\left(\mu -2\delta \right)\xi_{1}+\xi_{1}^{2}}$*. The PFP is LAS if* $n<\frac{d}{\xi_{2}}$ *and a saddle point if* $n>\frac{d}{\xi_{2}}$.

*Proof*: The Jacobian matrix of model (6) at ${ℰ}_{2}=\left(\frac{\Lambda }{\mu {ℛ}_{0}},\frac{\left({ℛ}_{0}-1\right)\Lambda }{\delta {ℛ}_{0}},0\right)$ is

$${𝒥(S,I,P)|}_{{ℰ}_{2}}=[\begin{array}{ccc} -\left(\mu +\xi_{1}\right) & -\delta & \Lambda k\\ \xi_{1} & 0 & -\xi_{2}m\\ 0 & 0 & \xi_{2}n-d \end{array}],$$ (15)

which gives eigenvalues: $\lambda_{1}=\xi_{2}n-d$ and $\lambda_{2,3}=-\frac{1}{2}\left[\mu +\xi_{1}\pm \xi_{3}\right]$. Since $\left|\mathrm{\backslash arg}\left(\lambda_{2,3}\right)\right|>\frac{\alpha \pi }{2}$ for any $\xi_{3}\in \mathbb{R}$, the local stability properties depend on the argument of $\lambda_{1}$. We compute that $\left|\mathrm{\backslash arg}\left(\lambda_{1}\right)\right|>\frac{\alpha \pi }{2}$ when $n<\frac{d}{\xi_{2}}$ and $\left|\mathrm{\backslash arg}\left(\lambda_{1}\right)\right|<\frac{\alpha \pi }{2}$ when $n>\frac{d}{\xi_{2}}$. Following Theorem 2, the LAS and saddle point properties given by Thoerem 9 are proven. □

**Theorem 10.** Let


$$c_{1}=\;(\mu +\beta \sigma )-\frac{am\sigma \kappa }{(1+\sigma a{)}^{2}},$$

$$c_{2}=\;\left(\beta \eta -\omega \right)\beta -\frac{(\mu +\beta \sigma )am\sigma \kappa }{(1+\sigma a{)}^{2}},$$

$$c_{3}=\;\left(\frac{\beta \Lambda k}{(1+\kappa k{)}^{2}}+\frac{(\mu +\beta \sigma )\sigma m}{1+\sigma a}\right)\frac{\kappa \sigma n}{(1+\sigma a{)}^{2}},$$

$$\Delta =\;\left(18c_{3}+c_{1}c_{2}\right)c_{1}c_{2}-\left(4\left(c_{1}^{3}c_{3}+c_{2}^{3}\right)+27c_{3}^{2}\right).$$

*The CEP* ${ℰ}_{3}=\left(\eta ,\sigma ,\kappa \right)$ *is LAS if the following statements hold.*

(i) Δ>0*, c*_1_>0*, c*_3_>0*, and* $c_{1}c_{2}>c_{3}$*, or*
(ii) Δ<0, $c_{1}\geq 0$, $c_{2}\geq 0$*, c*_3_>0 *and* α<2/3*, or*
(iii) Δ<0*, c*_1_>0*, c*_2_>0*, and* $c_{1}c_{2}=c_{3}$.

*If Δ<0, *c**_*1*_ *<0, *c**_*2*_ *<0, and α>2/3 then CEP is unstable.*

*Proof*: We obtain the Jacobian matrix at the CEP as follows.

$${𝒥(S,I,P)|}_{{ℰ}_{3}}=[\begin{array}{ccc} -(\mu +\beta \sigma ) & \omega -\beta \eta & -\frac{\Lambda k}{(1+\kappa k{)}^{2}}\\ \beta \sigma & \frac{am\sigma \kappa }{(1+\sigma a{)}^{2}} & -\frac{\sigma m}{1+\sigma a}\\ 0 & \frac{\kappa n}{(1+\sigma a{)}^{2}} & 0 \end{array}].$$ (16)

Thus, the polynomial characteristic of the Jacobian matrix (16) is $\lambda^{3}+c_{1}\lambda^{2}+c_{2}\lambda +c_{3}=0$. Using Generalized Routh-Hurwitz for Caputo fractional-order derivative as in Theorem 3, all statements given by Theorem 10 are well proven. □

### 4.4 Global stability analysis

We provide the global stability analysis of model (6) by employing the linear, quadratic, and Volterra function to construct the Lyapunov function as well as applying the generalized LaSalle invariance principle given by Lemmas 3 to 5. As a result, the following theorem is presented.

**Theorem 11.** *Let* $\tilde{S}=\frac{\Lambda }{\mu }$ *and hence* ${ℰ}_{1}=\left(\tilde{S},0,0\right)$*. The PDPF is GAS if* $\tilde{S}>\frac{\omega }{\beta }$ *and* $\frac{\Lambda kn}{dm}<{ℛ}_{0}<\frac{\beta \tilde{S}-\omega }{\beta \tilde{S}}$.

*Proof*: The model (6) can be written as

$${\;}^{C}{𝒟}_{t}^{\alpha }S=\;-\left(\mu +\beta I\right)\left(S-\tilde{S}\right)-\frac{\Lambda kP}{1+kP}-\left(\beta \tilde{S}-\omega \right)I,\;{\;}^{C}{𝒟}_{t}^{\alpha }I=\;\beta SI-\frac{\beta \tilde{S}}{{ℛ}_{0}}I-\frac{mIP}{1+aI},\;{\;}^{C}{𝒟}_{t}^{\alpha }P=\;\frac{nIP}{1+aI}-dP.$$ (17)

Now, we define a positive definite quadratic-linear Lyapunov function as follows.


$${𝒱}_{1}(x,y)=\frac{1}{2\tilde{S}}{\left(S-\tilde{S}\right)}^{2}+{ℛ}_{0}I+\frac{m{ℛ}_{0}}{n}P.$$

Using Lemma 3, the following inequality can be computed.


$${\;}^{C}{𝒟}_{t}^{\alpha }{𝒱}_{1}(x,y)\leq \;\frac{1}{\tilde{S}}\left(S-\tilde{S}\right){\;}^{C}{𝒟}_{t}^{\alpha }S+{\;}^{C}{𝒟}_{t}^{\alpha }I+\frac{m}{n}{\;}^{C}{𝒟}_{t}^{\alpha }P$$

$$=\;\frac{1}{\tilde{S}}\left(S-\tilde{S}\right)\left[-\left(\mu +\beta I\right)\left(S-\tilde{S}\right)-\frac{\Lambda kP}{1+kP}-\left(\beta \tilde{S}-\omega \right)I\right]$$

$$\;+{ℛ}_{0}\left[\beta SI-\frac{\beta \tilde{S}}{{ℛ}_{0}}I-\frac{mIP}{1+aI}\right]+\frac{m{ℛ}_{0}}{n}\left[\frac{nIP}{1+aI}-dP\right]$$

$$=\;-\frac{\mu +\beta I}{\tilde{S}}{\left(S-\tilde{S}\right)}^{2}-\frac{\Lambda kSP}{(1+kP)\tilde{S}}+\frac{\Lambda kP}{1+kP}$$

$$\;-\left(\beta -\left(\frac{\omega }{\tilde{S}}+\beta {ℛ}_{0}\right)\right)SI-\omega I-\frac{dm{ℛ}_{0}P}{n}$$

$$\leq \;-\frac{\mu +\beta I}{\tilde{S}}{\left(S-\tilde{S}\right)}^{2}+\Lambda kP-\left(\beta -\left(\frac{\omega }{\tilde{S}}+\beta {ℛ}_{0}\right)\right)SI$$

$$\;-\omega I-\frac{dm{ℛ}_{0}P}{n}$$

$$=\;-\frac{\mu +\beta I}{\tilde{S}}{\left(S-\tilde{S}\right)}^{2}-\left(\beta -\left(\frac{\omega }{\tilde{S}}+\beta {ℛ}_{0}\right)\right)SI-\omega I$$

$$\;-\left(\frac{dm{ℛ}_{0}}{n}-\Lambda k\right)P.$$

Using the fact $\frac{\Lambda kn}{dm}<{ℛ}_{0}<\frac{\beta \tilde{S}-\omega }{\beta \tilde{S}}$, we confirm that ${\;}^{C}{𝒟}_{t}^{\alpha }{𝒱}_{1}(x,y)\leq 0$. It also can be ensured that ${\;}^{C}{𝒟}_{t}^{\alpha }{𝒱}_{1}(x,y)=0$ only when $\left(S,I,P\right)=\left(\tilde{S},0,0\right)$. Obeying Lemma 5, the GAS properties of PDPF are justified. □

**Theorem 12.** *Let* $\hat{S}=\frac{\Lambda }{\mu {ℛ}_{0}}$, $\hat{I}=\frac{\left({ℛ}_{0}-1\right)\Lambda }{\delta {ℛ}_{0}}$*, and hence* ${ℰ}_{2}=\left(\hat{S},\hat{I},0\right)$*. The PFP is GAS if* $d>\frac{n}{m}\left(\frac{\beta \Lambda \hat{S}k}{\delta }+m\hat{I}+\frac{m}{a}\right)$

*Proof*: We rewrite model (6) into

$${\;}^{C}{𝒟}_{t}^{\alpha }S=\;-\frac{\Lambda kP}{1+kP}-\left(\mu +\beta I\right)\left(S-\hat{S}\right)-\delta \left(I-\hat{I}\right),\;{\;}^{C}{𝒟}_{t}^{\alpha }I=\;\left(\beta \left(S-\hat{S}\right)-\frac{mP}{1+aI}\right)I,\;{\;}^{C}{𝒟}_{t}^{\alpha }P=\;\frac{nIP}{1+aI}-dP.$$ (18)

We establish the positive definite Volterra-quadratic-linear Lyapunov function as follows.


$${𝒱}_{2}(x,y)=\frac{\beta }{2\delta }{\left(S-\hat{S}\right)}^{2}+\left(I-\hat{I}-\hat{I}\ln\frac{I}{\hat{I}}\right)+\frac{mP}{n}.$$

Using Lemmas 3 and 4, we obtain the following inequality.


$${\;}^{C}{𝒟}_{t}^{\alpha }{𝒱}_{2}(x,y)\leq \;\frac{\beta }{\delta }\left(S-\hat{S}\right){\;}^{C}{𝒟}_{t}^{\alpha }S+\frac{1}{I}\left(I-\hat{I}\right){\;}^{C}{𝒟}_{t}^{\alpha }I+\frac{m}{n}{\;}^{C}{𝒟}_{t}^{\alpha }P$$

$$=\;\frac{\beta }{\delta }\left(S-\hat{S}\right)\left(-\frac{\Lambda kP}{1+kP}-\left(\mu +\beta I\right)\left(S-\hat{S}\right)-\delta \left(I-\hat{I}\right)\right)$$

$$\;+\frac{1}{I}\left(I-\hat{I}\right)\left(\beta \left(S-\hat{S}\right)-\frac{mP}{1+aI}\right)I+\frac{m}{n}\left(\frac{nIP}{1+aI}-dP\right)$$

$$=\;-\frac{\beta \Lambda kSP}{(1+kP)\delta }+\frac{\beta \Lambda \hat{S}kP}{(1+kP)\delta }-\frac{\beta }{\delta }\left(\mu +\beta I\right){\left(S-\hat{S}\right)}^{2}$$

$$\;-\frac{mIP{\left(I-\hat{I}\right)}^{2}}{\left(1+aI)(1+a\hat{I}\right)}-\frac{mIP}{1+aI}+\frac{m\hat{I}P}{1+aI}+\frac{mIP}{1+aI}-\frac{dmP}{n}$$

$$\leq \;\frac{\beta \Lambda \hat{S}kP}{\delta }-\frac{\left(\mu +\beta I\right)\beta }{\delta }{\left(S-\hat{S}\right)}^{2}-\frac{mIP{\left(I-\hat{I}\right)}^{2}}{\left(1+aI)(1+a\hat{I}\right)}$$

$$\;+m\hat{I}P+\frac{mP}{a}-\frac{dmP}{n}$$

$$=\;-\frac{\left(\mu +\beta I\right)\beta }{\delta }{\left(S-\hat{S}\right)}^{2}-\frac{mIP{\left(I-\hat{I}\right)}^{2}}{\left(1+aI)(1+a\hat{I}\right)}$$

$$\;-\left(\frac{dm}{n}-\left(\frac{\beta \Lambda \hat{S}k}{\delta }+m\hat{I}+\frac{m}{a}\right)\right)P.$$

Since $d>\frac{n}{m}\left(\frac{\beta \Lambda \hat{S}k}{\delta }+m\hat{I}+\frac{m}{a}\right)$, we have ${\;}^{C}{𝒟}_{t}^{\alpha }{𝒱}_{2}(x,y)\leq 0$. We also have ${\;}^{C}{𝒟}_{t}^{\alpha }{𝒱}_{2}(x,y)=0$ only when $\left(S,I,P\right)=\left(\hat{S},\hat{I},0\right)$. Following Lemma 5, we confirm the PFP is GAS. □

**Theorem 13.** *Let*


$$\gamma_{1}=\;\frac{1}{2}\left(\omega +\sigma +\beta \gamma -\mu \right),$$

$$\gamma_{2}=\;\frac{1}{2}\left(\beta \gamma +\sigma +\eta \gamma -2\delta -\omega \right),$$

$$\gamma_{3}=\;\frac{1}{2a}\left(2n+(1+a)\kappa -2ad\right),$$

$$\gamma_{4}=\;\frac{1}{2}\left(\left(\mu +\beta \gamma \right)\eta^{2}+\sigma {\left(\delta +\omega \right)}^{2}+\sigma \omega^{2}+\beta^{2}\eta \gamma +\kappa d^{2}+\frac{n^{2}\kappa }{a}\right)$$

$$\;+\gamma \Lambda -\frac{\Lambda \eta }{1+\gamma k}.$$

*The CEP* ${ℰ}_{3}=\left(\eta ,\sigma ,\kappa \right)$ *is GAS if* $\gamma_{i}<0$ *for* i=1,2,3.

*Proof*: The model (6) can be rewritten as

$${\;}^{C}{𝒟}_{t}^{\alpha }S=\;\frac{\Lambda }{1+kP}-\left(\mu +\beta I\right)\left(S-\eta \right)-\left(\mu +\beta I\right)\eta +\omega \left(I-\sigma \right)+\omega \sigma ,\;{\;}^{C}{𝒟}_{t}^{\alpha }I=\;\beta I\left(S-\eta \right)-\left(\delta +\omega \right)\left(I-\sigma \right)-\frac{mIP}{1+aI}-\left(\delta +\omega \right)\sigma +\beta \eta I,\;{\;}^{C}{𝒟}_{t}^{\alpha }P=\;\frac{nI(P-\kappa )}{1+aI}-d\left(P-\kappa \right)-d\kappa +\frac{n\kappa I}{1+aI}.$$ (19)

Moreover, we construct a positive definite Volterra-quadratic Lyapunov function as follows.


$${𝒱}_{3}(x,y)=\frac{1}{2}{\left(S-\eta \right)}^{2}+\frac{1}{m}\left(I-\sigma -\sigma \ln\frac{I}{\sigma }\right)+\frac{1}{n}\left(P-\kappa -\kappa \ln\frac{P}{\kappa }\right),$$

which according to Lemmas 3 and 4, and the the upper bound *γ* given by Theorem 6, it can be derived as follows.


$${\;}^{C}{𝒟}_{t}^{\alpha }{𝒱}_{3}(x,y)\leq \;\left(S-\eta \right){\;}^{C}{𝒟}_{t}^{\alpha }S+\frac{1}{mI}\left(I-\sigma \right){\;}^{C}{𝒟}_{t}^{\alpha }I+\frac{1}{nP}\left(P-\kappa \right){\;}^{C}{𝒟}_{t}^{\alpha }P$$

$$=\;\left(S-\eta \right)\left[\frac{\Lambda }{1+kP}-\left(\mu +\beta I\right)\left(S-\eta \right)-\left(\mu +\beta I\right)\eta +\omega \left(I-\sigma \right)+\omega \sigma \right]$$

$$\;+\left(I-\sigma \right)\left[\beta I\left(S-\eta \right)-\left(\delta +\omega \right)\left(I-\sigma \right)-\frac{mIP}{1+aI}-\left(\delta +\omega \right)\sigma +\beta \eta I\right]$$

$$\;+\left(P-\kappa \right)\left[\frac{nI(P-\kappa )}{1+aI}-d\left(P-\kappa \right)-d\kappa +\frac{n\kappa I}{1+aI}\right]$$

$$=\;\frac{\Lambda S}{1+kP}-\frac{\Lambda \eta }{1+kP}-\left(\mu +\beta I\right){\left(S-\eta \right)}^{2}-\left(S-\eta \right)\left(\mu +\beta I\right)\eta$$

 +ω(S−η)(I−σ)+(S−η)ωσ+βI(S−η)(I−σ)

$$\;-\left(\delta +\omega \right){\left(I-\sigma \right)}^{2}-\frac{mIP}{1+aI}-\left(I-\sigma \right)\left(\delta +\omega \right)\sigma +\left(I-\sigma \right)\beta \eta I$$

$$\;+\frac{nI(P-\kappa {)}^{2}}{1+aI}-d{\left(P-\kappa \right)}^{2}-\left(P-\kappa \right)d\kappa +\frac{\left(P-\kappa \right)n\kappa I}{1+aI}$$

$$\leq \;\gamma \Lambda -\frac{\Lambda \eta }{1+\gamma k}-\left(\mu +\beta I\right){\left(S-\eta \right)}^{2}+\frac{\left(\mu +\beta I\right)}{2}{\left(S-\eta \right)}^{2}+\frac{\left(\mu +\beta \gamma \right)\eta^{2}}{2}$$

$$\;+\frac{\omega }{2}{\left(S-\eta \right)}^{2}+\frac{\omega }{2}{\left(I-\sigma \right)}^{2}+\frac{\sigma }{2}{\left(S-\eta \right)}^{2}+\frac{\sigma \omega^{2}}{2}+\frac{\beta \gamma }{2}{\left(S-\eta \right)}^{2}$$

$$\;+\frac{\beta \gamma }{2}{\left(I-\sigma \right)}^{2}-\left(\delta +\omega \right){\left(I-\sigma \right)}^{2}+\frac{\sigma }{2}{\left(I-\sigma \right)}^{2}+\frac{\sigma }{2}{\left(\delta +\omega \right)}^{2}$$

$$\;+\frac{\eta \gamma }{2}{\left(I-\sigma \right)}^{2}+\frac{\beta^{2}\eta \gamma }{2}+\frac{n}{a}(P-\kappa {)}^{2}-d{\left(P-\kappa \right)}^{2}+\frac{\kappa }{2}{\left(P-\kappa \right)}^{2}+\frac{\kappa d^{2}}{2}$$

$$\;+\frac{{\left(P-\kappa \right)}^{2}\kappa }{2a}+\frac{n^{2}\kappa }{2a}$$

$$=\;\gamma_{1}{\left(S-\eta \right)}^{2}+\gamma_{2}{\left(I-\sigma \right)}^{2}+\gamma_{3}{\left(P-\kappa \right)}^{2}+\gamma_{4}.$$

Since $\gamma_{i}<0,\;i=1,2,3$ affect that ${\;}^{C}{𝒟}_{t}^{\alpha }{𝒱}_{3}(x,y)\leq 0$ and ${\;}^{C}{𝒟}_{t}^{\alpha }{𝒱}_{3}(x,y)=0$ only when (S,I,P)=(η,σ,κ), we confirm from Lemma 5 that the CEP ${ℰ}_{3}$ satisfies GAS behavior. □

### 4.5 The existence of bifurcations

Bifurcations are a remarkable occurrence in mathematical modeling using fractional derivatives. In this subsection, we will show the occurrence of bifurcations which indicate the change of the dynamical behaviors when a parameter is varied. Several dynamics can occur such as the change of the number of equilibrium points, the change of the stability of the equilibrium points, and the occurrence of the limit cycle. The first bifurcation that occurs in this model is given by forward bifurcation. Forward bifurcation is a condition when a single stable (or unstable) equilibrium point changes into an unstable (or stable) equilibrium point accompanied by the occurrence of the new branch of stable (or unstable) equilibrium point [57,58]. The existence of forward bifurcation is given by the following theorem.

**Theorem 14.** *The PDPF undergoes a forward bifurcation when the basic reproduction number exceeds* ${ℛ}_{0}=1$.

*Proof*: It is clear that PDPF always exists. Furthermore, according to Theorems 7 and 8, when ${ℛ}_{0}<1$, the PDPF is LAS and the PFP does not exist. When ${ℛ}_{0}$ passes through ${ℛ}_{0}=1$, the PDPF becomes a saddle point while PFP is LAS. This ends the proof. □

Now, we identify the existence of the next bifurcation phenomenon called Hopf bifurcation. The occurrence of Hopf bifurcation was marked by the emergence of a limit cycle and the change of stability of an equilibrium point simultaneously driven by a parameter [59,60]. For the model with fractional derivative as the operator, the occurrence of Hopf bifurcation is not only driven by the parameters on the right-hand side of model (6) but also by the order of the derivative (α). Since the local stability of the equilibrium points lies on the argument of the eigenvalues $\lambda_{i},\;i=1,2,3$ of the Jacobian matrix evaluated at the equilibrium point, the Hopf bifurcation driven by *α* occurs by the following conditions [61,62]:

1. $\lambda_{1}<0$ and $\lambda_{2,3}=\theta \pm \omega i$ where θ>0;
2. $m(\alpha^{*})=\alpha^{*}\pi /2-\min_{1\leq i\leq 3}\left|\mathrm{\backslash arg}(\lambda_{i})\right|=0$;
3. ${\frac{dm(\alpha )}{d\alpha }|}_{\alpha =\alpha^{*}}\neq 0$.

If *α* passes through $\alpha^{*}=(2/\pi )\tan^{-1}(\omega /\theta )$, the stability of the equilibrium point change sign along by the occurrence of a stable limit cycle. In this condition, we call *α* the bifurcation parameter and $\alpha^{*}$ is the bifurcation point.

## 5 Numerical simulation

We provide some numerical simulations to support our analytical results, including giving new dynamical behaviors given by model (6) that cannot be described analytically. The Caputo Adam-Bashforth-Moulton (also called the generalized predictor-corrector) numerical scheme provided by Diethelm et al. [63] is employed to obtain the numerical solutions of the model. In this section, we identify the dynamical behaviors of model (6) when the fear level, infection rate, and memory index are varied. Since we do not study a specific ecological system that involves a specific species, all parameter values are provided hypothetically which is adjusted to analytical results. We provide them as follows.

**Fig 1:**
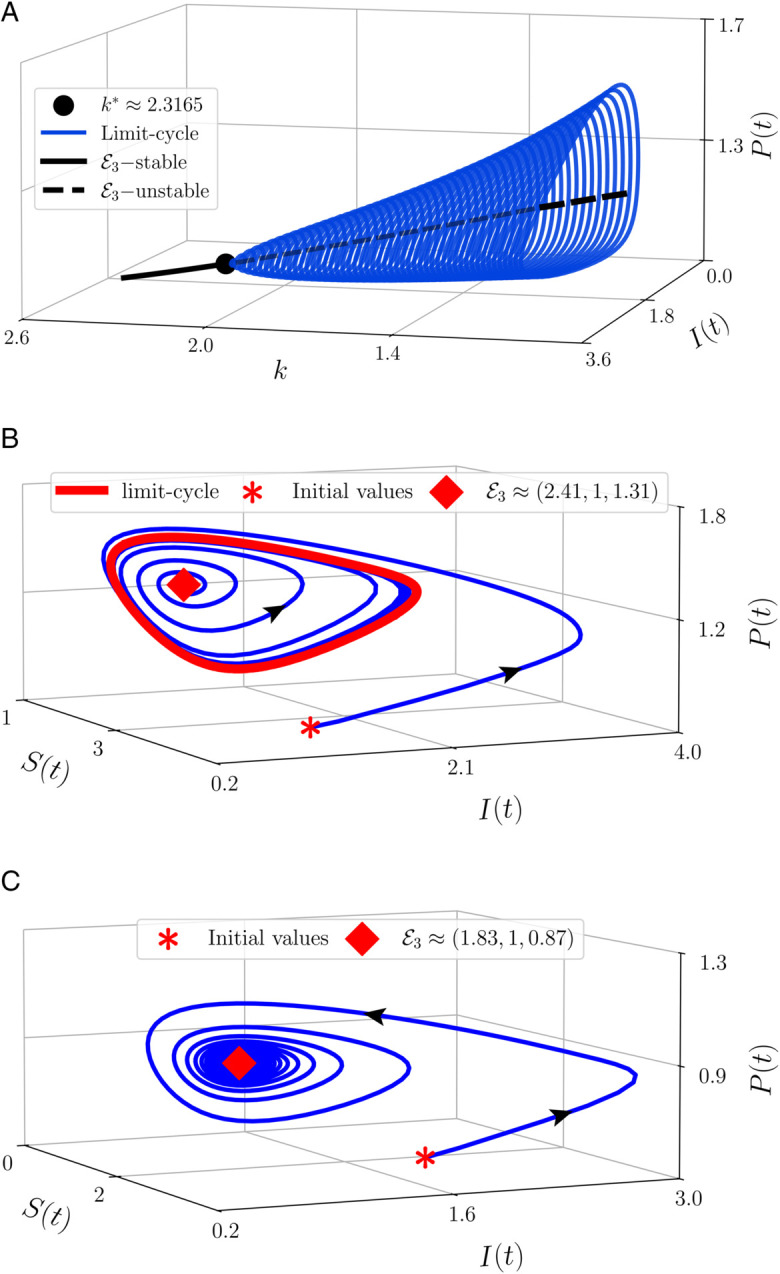
Bifurcation diagram and phase portraits of model (6) using parameter values: Λ = 10, *μ* = 0.5, *β* = 1.5, *ω* = 0.5, *δ* = 0.5, *m* = 4, *a* = 1, *n* = 1, *d* = 0.5, *α* = 0.95 and varying the value of *k.*

Now, we first investigate the dynamics of model (6) by studying the impact of fear. By setting the parameter values as in Table 2, and varying the fear level (*k*) in the interval [3,3.6], we have the bifurcation diagram and the phase portraits of model (6) around the CEP. In Fig 1(a), we find that when 1<*k* < *k*  for $k^{*}\approx 2.3165$, the solution in the interior of model (6) converges to the limit cycle while the CEP is unstable. Furthermore, if we increase the value of *k* passes through the critical point *k* , the CEP becomes asymptotically stable while the limit-cycle disappears. This circumstance indicates the occurrence of Hopf bifurcation. We confirm that the parameter *k* as the fear level becomes the bifurcation parameter while *k*  is the bifurcation point. From Fig 1(a) we also have a set of stable limit cycles for 1<*k* < *k* . The diameter of the limit cycle decreases when the value of *k* rises. To explore more, we portray the phase portrait of this condition at *k* = 1 and *k* = 2.5. When *k* = 1, we have Fig 1(b) which describes the condition when the solution near the CEP moves away from it and converges to the limit-cycle while the solution far from both the limit-cycle and CEP directly converges to the limit-cycle. When *k* = 2.5, these solutions converge to the CEP, and the limit cycle disappears. From the biological point of view, the given numerical simulations show the possibility of population density for susceptible prey, infected prey, and the predator when the fear level changes. If the fear level reduces, the density of all populations will change periodically with its period increasing. Conversely, if the effect of fear increases, the density of all populations will converge to an equilibrium point namely the co-existence point. All given simulations show that the density of all populations is always maintained in two different ways, one periodically and the other constantly converging to a single point.

**Table 2:** Parameter set of numerical simulations.

parameter	Λ	*k*	μ	β	ω	δ	*m*	*a*	*n*	*d*	α
values	10	0.4	0.5	1.5	0.5	0.5	4	1	1	0.5	0.95

**Fig 2:**
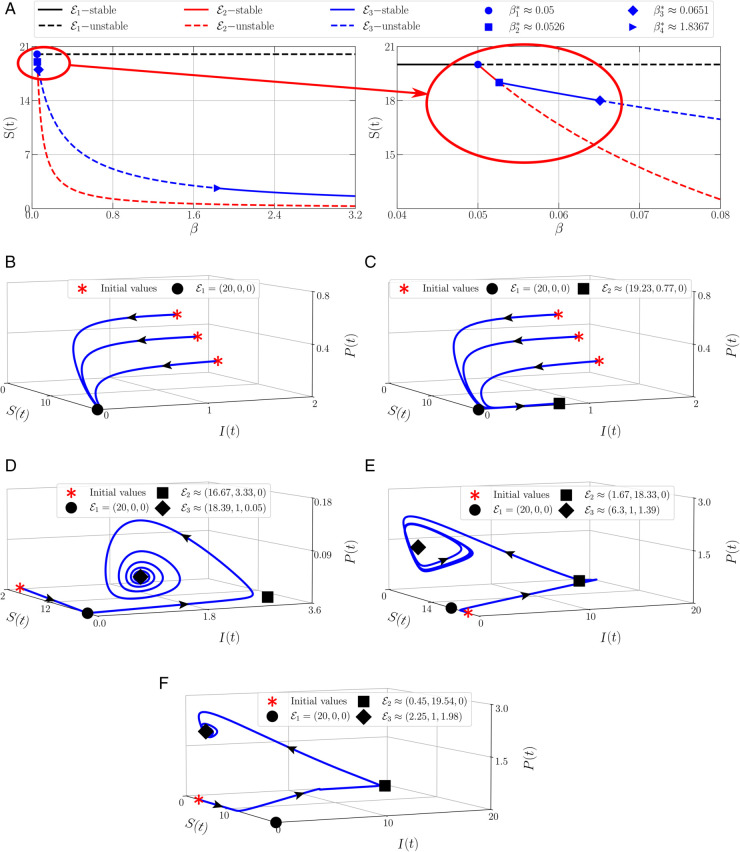
Phase portraits of model (6) using parameter values: Λ = 10, *k* = 0.4, *μ* = 0.5, *ω* = 0.5, *δ* = 0.5, *m* = 4, *a* = 1, *n* = 1, *d* = 0.5, *α* = 0.95, and varying the value of *β.*

For the next simulation, we investigate the impact of the disease on the existence of the populations. We set the parameter values as in Table 2 as well as varying the value of the infection rate (β). As a result, we have Fig 2(a) as the bifurcation diagram. For $0.04<\beta_{1}^{*}\approx 0.05$ $\left({ℛ}_{0}=1\right)$, only an asymptotically stable PDPF ${ℰ}_{1}$ exists while other does not exist. We set β=0.04 and hence ${ℛ}_{0}=0.8<1$ which is by the analytical result that the PDFP is locally asymptotically stable, see the phase portrait given by Fig 2(b). It shows that the solutions converge to ${ℰ}_{1}=(20,0,0)$. Furthermore, when *β* crosses $\beta_{1}^{*}$, the PDPF loses its stability via forward bifurcation and a new branch of an asymptotically stable equilibrium point occurs namely the PFP. The stability of PFP is maintained for interval $\beta_{1}^{*}<\beta <\beta_{2}^{*}\approx 0.0526$ $\left({ℛ}_{0}=1.052\right)$. If we set β=0.052, we obtain Fig 2(c) as the phase portrait which shows that the PDPF ${ℰ}_{1}=(20,0,0)$ becomes a saddle point while the PFP ${ℰ}_{2}\approx (19.23,0.77,0)$ is asymptotically stable. Similarly, the PFP also loses its stability via forward bifurcation and an asymptotically stable CEP occurs simultaneously when *β* crosses $\beta_{2}^{*}$. This condition holds for $\beta_{2}^{*}<\beta <\beta_{3}^{*}\approx 0.0651$ $\left({ℛ}_{0}=1.302\right)$, For example, we choose β=0.06 and hence we have Fig 2(d). The PDPF and PFP become saddle points and the solution converges to the CEP ${ℰ}_{3}\approx (18.39,1,0.05)$. When *β* passes through $\beta_{3}^{*}$, the PFP loses its stability via Hopf bifurcation and the solution will converge to a limit-cycle. We set β=0.6 and we can see from Fig 2(e) that a limit-cycle occurs as the impact of Hopf bifurcation while the PFP ${ℰ}_{3}\approx (6.3,1,1.39)$ unstable. The instability of CEP is maintained for $\beta_{3}^{*}<\beta <\beta_{4}^{*}\approx 1.8367$ $\left({ℛ}_{0}=36.734\right)$. Again, a Hopf bifurcation occurs as a result of the increase in infection rate. When *β* crosses $\beta_{4}^{*}$, the limit cycle disappears and the CEP becomes an asymptotically stable equilibrium point, see Fig 2(f) for the example of the phase portrait which shows that the solution converges to ${ℰ}_{3}\approx (2.25,1,1.98)$. From those simulations given by Fig 2, we conclude that the infection rate has a global impact on all populations. For the ${ℛ}_{0}<1$, the infected prey and the predator will become extinct, where only the susceptible prey exists in the ecosystem. If the infection rate increases, the infected prey occurs and the predator extinct. When the infection rate increases again, the susceptible prey, infected prey, and predator will always exist in the ecosystem in two ways, directly tend to a co-existence point or change periodically around the co-existence point. It means the disease impacts not only the prey’s existence but also the predator’s existence.

**Fig 3:**
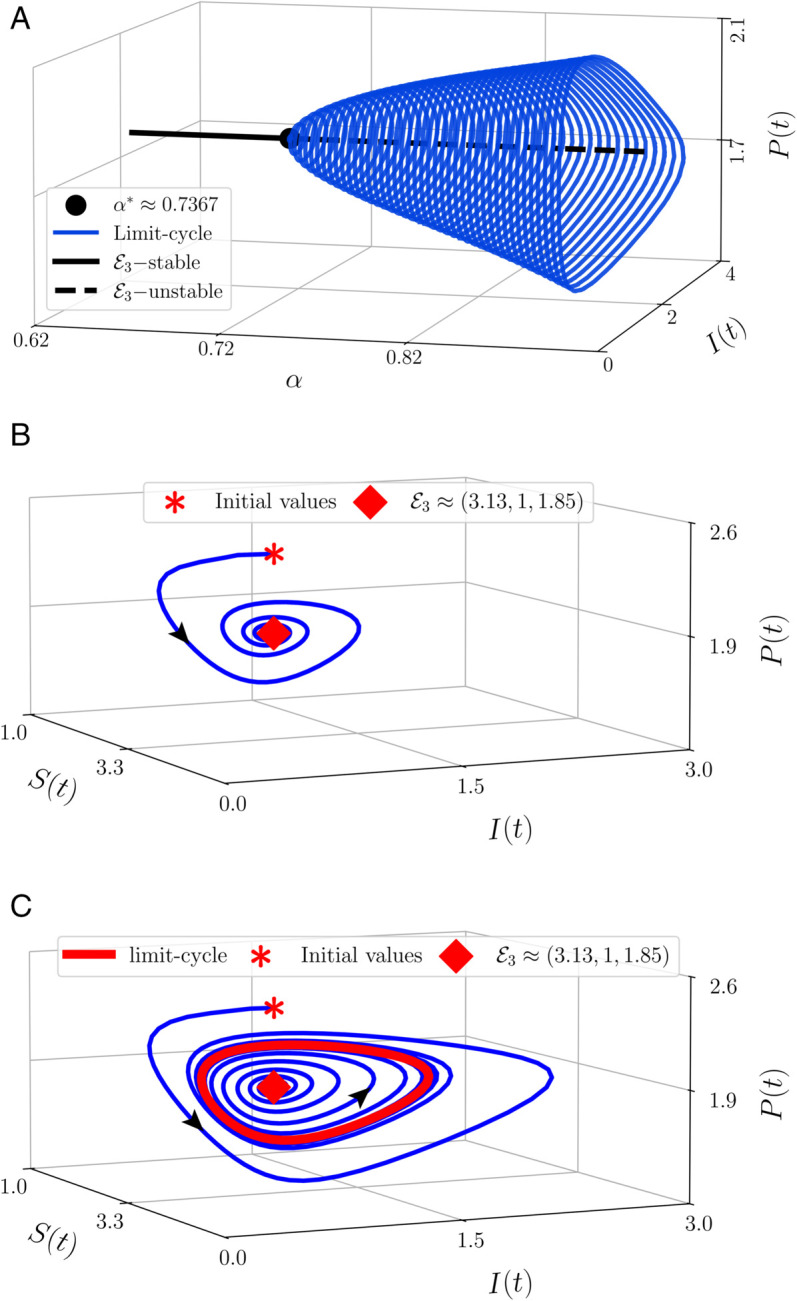
Phase portraits of model (6) using parameter values: *λ* = 10, *k* = 0.4, *μ* = 0.5, *β* = 1.5, *ω* = 0.5, *δ* = 0.5, *m* = 4, *a* = 1, *n* = 1, *d* = 0.5, and varying the value of *α.*

The final numerical simulations are done to show the dynamical behaviors of model (6) by considering the index of the memory which is stated by the order of the derivative (α). The Table 2 are used as the parameter values. By varying the order in the interval 0.62≤α≤1, we obtain the bifurcation diagram given by Fig 3(a). It shows that when $0.62\leq \alpha <\alpha^{*}\approx 0.7367$, the CEP ${ℰ}_{3}$ is asymptotically stable. When *α* crosses $\alpha^{*}$, a set of limit cycles occurs until α=1 and the CEP becomes unstable. The diameter of the limit cycle also increases when *α* rises. This phenomenon is called Hopf bifurcation where *α* is the bifurcation parameter and $\alpha^{*}$ is the bifurcation point. Biologically, this circumstance indicates that the memory index affects the dynamics of the population. For a strong memory, the susceptible prey, infected prey, and the predator will directly converge to a constant population point and successfully maintain their existence. For a weak memory, the all population also gets their existence but in different ways. Each of them will eventually change periodically without losing their existence.

## 6 Conclusions

The dynamical behaviors of model (6) which represent an interaction between prey and its predator have been studied. Some assumptions are provided to include more realistic phenomena in the model such as the fear factor, a disease in prey, and the memory effect. The mathematical validity has been given by showing the existence, uniqueness, non-negativity, and boundedness of the model. The dynamics of the model have been studied both analytically and numerically. We have found three equilibrium points along with their dynamical behaviours both locally and globally by applying the Matignon condition, Lyapunov function, and LaSalle invariance principle. The occurrence of Hopf bifurcation driven by the memory index has been proven analytically. The dynamical behaviors have been finally explored numerically by showing the occurrence of forward and Hopf bifurcation driven by the fear level, infection rate, and memory effect using the bifurcation diagrams under the generalized Adam-Bashforth-Moulton numerical scheme. To support all results, some phase portraits are given for each interval in bifurcation diagrams which describe the change of the dynamical behaviors when the bifurcation parameter passes through the bifurcation point. From the biological point of view, some scenarios may occur such as the extinction of both infected prey and predator, the extinction of predator only, or the existence of all populations by maintaining their population densities in two ways, directly converging to a single point or will eventually change periodically. Although the model is completelly analyzed both analytically and numerically, we can develop the model by considering the real phenomena given by a specific species by considering their unique biological components, for example the way the predator hunting the prey, the prey defence, the climate change, and so forth. These conditions will give a significant impact to the mathematical model as well as the results.
